# Change in surface properties of two different dental resin composites after using various beverages and brushing

**DOI:** 10.1186/s12903-023-03710-8

**Published:** 2023-12-05

**Authors:** Lamia M. Elmalawany, Dina A. El-Refai, Ghada A. Alian

**Affiliations:** Biomaterials Department, Faculty of Dentistry, Ain-Shams University, African Union Organization Street, Abbasia, Cairo 11566 Egypt

**Keywords:** Surface hardness, Surface roughness, Ormocer, Nano-hybrid resin composite, Brushing, Beverages

## Abstract

**Background:**

The study aimed to evaluate the influence of various beverages; with and without brushing; on the surface mechanical properties of two resin composites.

**Methods:**

A total of 160 disc-shaped specimens were prepared for each of the following dental composites; nanohybrid ormocer (Admira fusion, VOCO GmbH, Cuxhaven, Germany) and nanohybrid resin composite (Grandio, VOCO GmbH, Cuxhaven, Germany). The baseline surface hardness and roughness measurements were carried out after 24 h. The composite samples were randomly distributed into one of the two groups; brushing and non-brushing, which were further divided into one of the four subgroups (*n* = 10); artificial saliva as control, coffee, red wine, and soft drink. In the non-brushing group, the specimens were immersed in the different beverages for five minutes three times daily for 30 days. The same procedure was done for the brushing group, in addition to brushing the specimens for five seconds. The surface hardness and roughness measurements were repeated after 30 days. One-way ANOVA and independent t-tests were used for statistical analysis.

**Results:**

The soft drink had the most deteriorating effect and artificial saliva had the least. The change in surface properties was higher in the brushing subgroups. Grandio exhibited a higher change in surface microhardness while Admira fusion exhibited a higher change in surface roughness.

**Conclusions:**

The surface properties of both dental resin composites were negatively affected by using beverages and brushing.

## Background

Dental resin composites are among the most frequently used dental materials for esthetic restorations in dental practice [1]. This is attributed to their ability to bond to enamel and dentine, resemblance to tooth structures in color and mechanical properties, ease of chair-side applications as well as their relatively low cost [2].

Continuous developments have been made in dental resin composite composition to achieve better esthetic and mechanical properties in terms of filler loading, filler size, and matrix modification [3]. One of these developments is the ormocer which is the acronym for organically modified ceramic [3]. This technology combines organic and inorganic components at a nanoscopic scale through the sol–gel method [4]. Moreover, further advancements have been made to the ormocer composite with Admira Fusion (VOCO GmbH), being introduced to the market, as the world's first pure ceramic‐based restorative material without the addition of conventional dimethacrylate [5].

The surface characteristics of composite resins are crucial for their clinical success, as rough surfaces might cause discoloration, plaque accumulation, recurrent caries, and gingival irritation, in addition to producing inconvenience during cleaning procedures [6].

The surface hardness of dental resin composites can give an indication of the degree of conversion and consequently the clinical performance of resin composite material after aging in food-simulating solvents [7].

Despite that, dental resin composites must survive an aggressive oral environment that varies from one patient to another as do the masticatory forces, occlusal habits, chemically active foods and liquids, temperature fluctuations, humidity variations, bacterial products, as well as salivary enzymes. These factors, separately or collectively, determine the longevity of the restoration [7].

The popularity of different beverages with high acidic or alcoholic content has raised questions about their degradative potential [8]. Acidic and alcoholic content present in different beverages can cause a reduction in the surface microhardness of composites by softening the bis-GMA based polymers present in the organic matrix [8, 9].

To counteract the staining effect of these beverages, brushing is recommended to remove the superficial staining partially or even completely and enhance the color stability of dental resin composite restorations [10, 11]. Despite that, toothbrush abrasion can cause esthetic and biological disadvantages in the long term, such as decreased gloss, discoloration and/or staining of the material surface, and increased accumulation of dental plaque [12]. Brushing can cause degradation of the polymer matrix of the composite resin thus changing the surface hardness of the composite resin, which subsequently enhances more discoloration [13–15]. This is influenced by the abrasiveness of the toothpaste which is referred to as Relative Dentin Abrasion (RDA) [16]. The RDA of whitening toothpastes ranges either between 60 and 100 or higher than 100 [6]. It has been claimed that using toothpaste with high abrasiveness would increase the surface roughness [16].

The null hypothesis of this study was that there would be no change in the surface microhardness and roughness of the nano-hybrid composite resin and nano-hybrid ormocer immersed in different beverages; with and without brushing.

## Methods

### Materials

Materials used in this study are shown in Table 1.

**Table 1:** Materials used in the study

Brand name	Description	Composition	Manufacturer	Lot no
**Dental resin composites used:**				
**Admira Fusion (A3)**	Universal nanohybrid-ORMOCER	100% ormocer monomer with C = C groups matrix 84 wt% inorganic filler loading	VOCO GmbH, Cuxhaven, Germany	1706603
**Grandio (A3)**	Universal nanohybrid resin composite	Bis-GMA, TEDGMA, UDMA matrix 87 wt% / 71.4 Vol.% inorganic filler loading	VOCO GmbH, Cuxhaven, Germany	1705400
**Beverages used:**				
**Artificial saliva**		4.1 mM KH_2_PO_4_, 4.0 mM Na_2_HPO_4_, 24.8 mM KHCO_3_, 16.5 mM NaCl, 0.25 mM MgCl_2_, 4.1 mM citric acid and 2.5 mM CaCl_2._ The pH of the artificial saliva solution was adjusted to 6.7with 10 N HCl ^(99)^	Laboratory of the pharmaceutical industry	
**Nescafe Classic**	Instant coffee	100% pure soluble coffee made of robusta coffee beans through spray drying technique	Packed by Nestle, Egypt Made in Spain	
**Red wine**	Alcoholic drink	Sugar-free red wine, alcohol content 12.5% Vol	Gianclis Vineyards, Egypt	
**Pepsi Cola**	Soft drink	Carbonated water, Sugar or Fructose syrup, Color (caramel), Phosphoric acid, Caffeine, Emulsifier (gum Arabic), Natural flavor	Pepsi Cola, Egypt	
**Dentifrice used:**				
**Signal Complete 8 white (RDA = 140)**		Sodium Fluoride (1450 ppm Fluoride), Zinc Citrate, Aqua (water), Sorbitol, Hydrated Silica, PEG-32, Sodium Lauryl Sulfate, Aroma (flavor), Cellulose Gum, Perlite, Sodium Fluoride, Sodium Saccharin, Mica, Glycerin, Cl 74160 & Cl 77891	Unilever Mashreq, Egypt	ABN07

### Sample size calculation

The sample size was calculated using G power for sample size analysis at a power of 80%, a significant level of 5%, and large effect size (0.4) which yields a total sample size of 160; 10 samples per subgroup.

### Specimens’ grouping

**Fig. 1:**
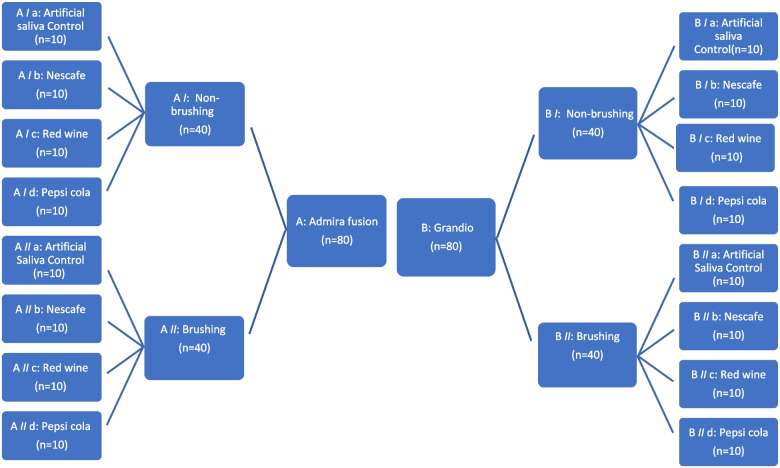
Flow chart showing Admira Fusion and Grandio specimens’ grouping for each test

One hundred and sixty specimens from each composite type were prepared; eighty for each test. Specimens from each dental resin composite type were divided into two main groups (40 specimens each) according to non-brushing or brushing procedure. Then each group was further subdivided into four subgroups according to the used beverages (*n* = 10) (Fig. 1).

### Specimen preparation

Split Teflon mold having a central circular hole of dimensions 10 mm diameter and 2 mm thickness was used to prepare the specimens [1]. First, a celluloid matrix was placed over a glass microscopic slide then the mold was placed over it. The composite was placed as a single increment and covered with a celluloid matrix and another glass slide. To obtain a flat surface and to standardize the force applied to the surface, a weight of 500 g was placed over the glass slide for 1 min. After the removal of the weight, the specimen was light cured through the glass slide by light-emitting diode (LED) light cure of 1200 mW/cm2 output (Elipar S10, 3M ESPE, Germany), which was periodically checked using a radiometer (Model 100 curing radiometer, Kerr, USA), for 20 s according to the manufacturer’s instructions. The prepared specimens were stored in artificial saliva for 24 h in an incubator at 37℃ [5].

### Surface microhardness testing

The baseline microhardness measurements were recorded using a digital Vickers hardness tester (Nexus 4000 TM, INNOVATEST, model no. 45.3, Netherlands). For each specimen, the bottom surface was marked so that measurements were done on the top surface. Three indentations were done for each specimen using a 300 g load for 10 s [17] and examined at 20X magnification. The indentations were done as follows; one indentation in the center of the specimen and the other two indentations were to the right and the left of the central one, guided by marks done at the bottoms of the specimens. The average of the three readings was taken and microhardness value was calculated for each specimen.

These measurements were repeated at the end of the immersion period (30 days) [6] in different beverages and the post-immersion readings were calculated. Then the pre- and post-immersion readings were compared for each specimen.

### Surface roughness testing

A surface profilometer (TR 220 Surface Roughness Tester, TIME Group, Pittsburgh, PA, USA) was used to measure the surface roughness, with a cut-off value of 0.25 mm. A total of three measurements were taken. The measures were made as follows: one measurement was taken in the specimen's center, and the other two measurements were taken to the right and left of the central measurement. For each specimen, the average surface roughness (Ra) was calculated.

These measurements were repeated at the end of the immersion period (30 days) in different beverages and the post-immersion readings were calculated. Then the pre- and post-immersion readings were compared for each specimen.

### Immersion cycles and brushing

Specimens were then immersed in the various beverages for 5 min [18] 3 times daily with a time interval of 5 h. In between the cycles, the specimens were kept in artificial saliva at 37℃ in an incubator (Titanox, Italy). Beverages were used according to the common temperature of consumption i.e., Pepsi Cola 4 ± 1℃ (kept in a refrigerator), red wine 25 ± 1℃ (kept in an incubator), and Nescafe was prepared by adding 5 gm of powder to 250 ml of boiling distilled water and stirred for 1 min using cappuccino mechanical stirrer and cooled to 70 ± 1℃. The temperature was checked using a digital thermometer. Freshly prepared beverages were used for each immersion cycle. After each immersion cycle, the specimens were washed with distilled water for 1 min, blot-dried, and returned to the artificial saliva. The artificial saliva was renewed daily, and the procedure was repeated for 30 days [6, 15, 18].

For the brushing group, the same procedure was done. Then brushing was done using an electric brush (Oral-B Vitality, Braun Gmbh, Germany) one hour after the last cycle. The dentifrice was used in the form of a slurry by mixing the paste with distilled water in a ratio of 1:1 by volume [10] which was then applied to the top surface of the specimen using a spatula. Then the brushing was carried out for 5 s [10] with an applied vertical load of 200 g. A new brush was used for each subgroup.

After 30 days of immersion in the recommended beverages, specimens were submitted to the microhardness, and surface roughness testing. Post-immersion measurements were recorded. The change in the microhardness and surface roughness values of each specimen was calculated.

### Statistical analysis

Values were presented as mean, standard deviation (SD) standard error (SE) values. Data were explored for normality using the Kolmogorov–Smirnov test of normality. The results of the Kolmogorov–Smirnov test indicated that most of the data were normally distributed (parametric data), therefore, three-way ANOVA was used to study the interaction of the 3 variables.

One-way analysis of variance (ANOVA) test was used between subgroups within each group for each material. This was followed by Tukey’s post hoc test when ANOVA yielded a significant difference. An independent t-test was used to compare the corresponding sub-groups of the two groups for each material and between corresponding sub-groups for both materials together.

The significance level was set at *p* < 0.05. Statistical analysis was performed with SPSS 18.0 (Statistical Package for Scientific Studies, SPSS, Inc., Chicago, IL, USA) for Windows.

## Results

### Surface hardness results

The three-way ANOVA showed that regarding the change in surface microhardness values in different beverages, there was a statistically significant difference (*p* < 0.001) among the tested subgroups. Regarding the change in surface microhardness values of different materials, there was a statistically significant difference (*p* < 0.001) among the tested materials. Regarding the change in surface microhardness values with/without brushing, there was a statistically significant difference (*p* < 0.001) among the tested groups. The interaction of material and different beverages variables had a statistically significant effect (*p* < 0.001). Also, the interaction of brushing and different beverages variables had a statistically significant effect (*p* < 0.001). The interaction of material & brushing had a statistically significant effect (*p* < 0.001). In addition, the interaction of the 3 variables had a statistically significant effect (*p* < 0.001).

Pre-immersion and post-immersion values of Vickers microhardness number (VHN) were recorded and the change in the microhardness (Δ VHN) was calculated for each specimen.

Means, standard deviations, and standard errors of the change in microhardness values (Δ VHN) for each type of dental resin composite after being subjected to different beverages used in the study, with/without brushing, were presented in Table 2.

**Table 2:** Means, SD, and SE of the change in microhardness values (Δ VHN) for each type of dental resin composite after being subjected to different beverages used in the study with/without brushing regarding the effect of different types of dental resin composites

Material	Group	Beverages	Mean	SD	SE	*p*-value
Admira fusion	Non-Brushing	Artificial saliva (Control)	-2.60^c^	1.67	.75	0.00*
		Nescafe Classic	-5.20^b^	.84	.37	
		Red wine	-8.20^a^	.84	.37	
		Pepsi Cola	-9.50^a^	.61	.27	
	Brushing	Artificial saliva (Control)	-10.60^b^	.89	.40	0.00*
		Nescafe Classic	-10.20^b^	.67	.30	
		Red wine	-12.70^a^	.57	.25	
		Pepsi Cola	-11.90^a^	.74	.33	
Grandio	Non-Brushing	Artificial saliva (Control)	-5.60^c^	2.41	1.08	0.00*
		Nescafe Classic	-9.70^b^	.97	.44	
		Red wine	-16.40^a^	1.52	.68	
		Pepsi Cola	-15.90^a^	1.75	.78	
	Brushing	Artificial saliva (Control)	-10.20^c^	.84	.37	0.00*
		Nescafe Classic	-13.30^b^	1.20	.54	
		Red wine	-6.00^d^	.79	.35	
		Pepsi Cola	-15.20^a^	.67	.30	

Significance level *P* < 0.05, *significant

Tukey’s post hoc test: means sharing the same superscript letters are not significantly different

When comparing the effect of the different beverages, for the Admira fusion, in the non-brushing group, the highest Δ VHN mean value was recorded in the subgroup using Pepsi cola (-9.50), whereas the lowest mean value was recorded in the control subgroup (-2.60). In the brushing group, the highest Δ VHN mean value was recorded in the subgroup using red wine (-12.70), whereas the lowest mean value was recorded when immersed in Nescafe Classic (-10.20).

For the Grandio in the non-brushing group, the highest Δ VHN mean value was recorded in the subgroup using red wine (-16.40), whereas the lowest mean value was recorded in the control subgroup (-5.60). In the brushing group, the highest Δ VHN mean value was recorded in the subgroup using Pepsi Cola (-15.20), whereas the lowest mean value was recorded on exposure to red wine (-6.00).

When comparing the brushing and non-brushing groups, for the Admira Fusion within all beverages, brushing revealed the highest Δ VHN mean values with a significant difference between the brushing and non-brushing groups. While for the Grandio within all beverages, the brushing group yielded the highest Δ VHN mean values except for using red wine.

When comparing the two materials no significant difference was found between them when stored in artificial saliva with/without brushing.

When immersed in Nescafe Classic, the highest Δ VHN mean value was recorded with Grandio, with a significant difference (*p* < 0.001) compared to Admira fusion with/without brushing.

When immersed in red wine, in the non-brushing group, the highest Δ VHN mean value was recorded with Grandio, with a significant difference (*p* < 0.001) compared to Admira fusion. In the brushing group, the highest Δ VHN mean value was recorded in Admira fusion, with a significant difference (*p* < 0.001) compared to Grandio.

When immersed in Pepsi Cola, the highest Δ VHN mean value was recorded with Grandio, with a significant difference (*p* < 0.001) compared to Admira fusion with/without brushing.

### Surface roughness results

The three-way ANOVA showed that regarding the change in surface roughness in different beverages, there was a statistically significant difference (*p* < 0.001) among the tested subgroups. Regarding the change in surface roughness of different materials, there was a statistically significant difference (*p* < 0.001) among the tested materials. Regarding the change in surface roughness with/without brushing, there was a statistically significant difference (*p* < 0.001) among the tested groups. The interaction of material and different beverages variables had a statistically significant effect (*p* < 0.001). Also. the interaction of brushing and different beverages variables had a statistically significant effect (*p* < 0.001). The interaction of material & brushing had a statistically significant effect (*p* < 0.001). In addition, the interaction of the 3 variables had a statistically significant effect (*p* < 0.001).

Pre-immersion and post-immersion values of surface roughness (Ra) were recorded and the change in the surface roughness (Δ Ra) was calculated for each specimen.

Means, standard deviations, and standard errors of the change in surface roughness values (Δ Ra) for each type of dental resin composite after being subjected to different beverages used in the study, with/without brushing, were presented in Table 3.

**Table 3:** Means, SD, and SE of the change in surface roughness (Δ Ra) for each type of dental resin composite after being subjected to different beverages used in the study with/without brushing regarding the effect of different beverages

Material	Group	Beverages	Mean	SD	SE	*p*-value
Admira fusion	Non-Brushing	Artificial saliva (control)	.004^c^	.002	.001	0.00*
		Nescafe Classic	.005^c^	.002	.001	
		Red wine	.014^b^	.003	.001	
		Pepsi Cola	.020^a^	.002	.001	
	Brushing	Artificial saliva (control)	.009^d^	.001	.001	0.00*
		Nescafe Classic	.016^c^	.001	.000	
		Red wine	.029^b^	.001	.001	
		Pepsi Cola	.065^a^	.003	.001	
Grandio	Non-Brushing	Artificial saliva (control)	.005^a^	.001	.000	0.187 ns
		Nescafe Classic	.004^a^	.001	.000	
		Red wine	.004^a^	.001	.000	
		Pepsi Cola	.003^a^	.001	.001	
	Brushing	Artificial saliva (control)	.008^c^	.001	.001	0.00*
		Nescafe Classic	.020^b^	.003	.001	
		Red wine	.019^b^	.003	.001	
		Pepsi Cola	.024^a^	.001	.001	

Significance level *P* < 0.05, *significant

Tukey’s post hoc test: means sharing the same superscript letters are not significantly different

When comparing the effect of the different beverages, for the Admira Fusion with/without brushing, the highest Δ Ra mean value was recorded in the subgroup immersed in Pepsi Cola, whereas the lowest value was recorded with the control subgroup.

For the Grandio in the non-brushing group, the highest Δ Ra mean value was recorded in the control subgroup (0.005), whereas the lowest value was recorded using Pepsi Cola (0.003). In the brushing group, the highest Δ Ra mean value was recorded in Pepsi Cola (0.024), whereas the lowest value was recorded in the control subgroup (0.008).

When comparing the brushing and non-brushing groups, for both Admira Fusion and Grandio within all beverages, brushing revealed the highest Δ Ra mean value with a significant difference between brushing and non-brushing groups.

When comparing the two materials no significant difference was found between them when stored in artificial saliva with/without brushing.

When immersed in Nescafe Classic, in the non-brushing group, there was no significant difference between the two materials. In the brushing group, the highest Δ Ra mean value was recorded in Grandio, with a significant difference (*p* = 0.039) compared to Admira fusion.

When immersed in red wine with/without brushing, the highest mean value Δ Ra was recorded with Admira fusion, with a significant difference compared to Grandio.

On using Pepsi Cola, with/without brushing, the highest mean value Δ Ra was recorded with Admira fusion, with a significant difference compared to Grandio.

## Discussion

Consuming certain beverages, such as coffee, alcoholic beverages, and cola drinks, may have an impact on the esthetic and physical characteristics of resin composites, which could lower the restoration's quality [19]. The effect of these beverages on the surface microhardness and roughness of resin composites varies depending on the intrinsic features of the composite such as their chemical composition. In addition to that, brushing can have a great effect on the degradation of surface properties of dental resin composites.

Thus, the conducted study aimed to assess the effect of different beverages, with/without brushing, on the surface mechanical properties of dental resin composite. The null hypothesis was rejected as the various beverages with/without brushing caused a change in surface microhardness and roughness of the two dental resin composites.

One factor affecting the surface quality of dental resin composites is the finishing and polishing procedure. It has been reported that the microhardness of a celluloid strip finished composite surface was lower than the composite itself. However, finishing the composite surface with a celluloid strip can produce the smoothest resin composite surface [20]. In this study, the composite samples were light-cured in contact with a celluloid strip to eliminate the influence of the variability of finishing techniques on the results.

Food and drink only briefly contact the tooth surfaces while being consumed before being rinsed away by saliva. Previous studies often involved substrates coming into prolonged contact with acidic food substances, which is unrepresentative of clinical situations and does not take the washing role of saliva into account. For this reason, a cycle of 5 min of immersion was used in this study, and it was repeated three times daily to mimic the clinical situation.

While a person may wash their teeth for two minutes, only a portion of that time is likely spent actually scrubbing each tooth surface. Approximately four seconds should be spent brushing each tooth each day [21]. The specimens in our investigation were brushed for 5 s.

Regarding the surface microhardness results, the change in surface microhardness was the highest upon immersion in Pepsi Cola and red wine, and it was the least in the control subgroup. This was in agreement with Wongkhantee et al., [22] who reported that during a short period of contact (simulating drinking a can of soft drink), Cola significantly reduced surface hardness of enamel, dentine, micro-filled composite, and resin-modified glass ionomer. Also, Nazish et al., [23] reported a decrease in surface hardness of resin composites when exposed to acidic media. Cavalcantea et al., [24] observed that the immersion in ethanol induced a higher decrease in surface hardness values in all materials used in their study and attributed this reduction in microhardness to the higher amount of [–OH] present in the ethanol, so a higher absorption occurs by the polar portion of the matrix, causing swelling of the material. This dimensional change in the matrix causes stress at the matrix–silane–filler particle interfaces, resulting in the degradation of the bond [25]. In addition to that, soft drinks with an acidic nature can cause a reduction in the surface microhardness of composites by softening the Bis-GMA-based polymers present in the organic matrix [1]. As these solvents diffuse into the network system of the polymer it causes expansion and loss of the unreacted monomers, oligomers, and ions. Consequently, these solvents occupy the porosities and act as plasticizers without forming any chemical bonds to the network system thus reducing the hardness [7]. As a probable consequence, the inorganic particles are no longer provided with a stable structure, which could predispose to filler dislodgment and elution [7].

The examination of the beverage's erosive potential requires consideration of its chemical properties, including pH, titratable acidity, and buffering capacity [26, 27]. Although all the used staining solutions have an acidic nature, Pepsi cola had the lowest initial pH and the highest titratable acidity and buffering capacity which increased its erosive ability [28].

Vouvoudi and Sideridou [29] reported that storage in water or artificial saliva at 37 ℃ for 1 or 7 days caused post-curing reactions, while storage for 30 or 90 days seems to cause plasticization effect affecting some parameters analogously. This could explain why the control subgroup showed a reduction in microhardness even if it was the least.

Brushing yielded the highest change in surface microhardness. There was clear evidence that in the presence of an abrasive, resin composites are susceptible to micro-scale abrasion depending on the polymeric matrix and inorganic fillers. This behavior is mainly due to the lower hardness of the exposed polymeric matrix when compared to inorganic particles. The same kind of wear response is expected to occur in dental applications involving these materials due to the micro-scale abrasion action promoted by the hard particles present in food or toothpaste [12].

The change in surface microhardness was less obvious in Admira fusion in most tested groups which is in agreement with Moyin et al. [9]. This could be attributed to the nature of Admira fusion, being an ormocer. Ormocers are basically organically modified ceramic with poly-condensed organic–inorganic networks. This new class of material combines the surface properties of the silicones, the toughness of the organic polymers, and the hardness and thermal stability of ceramics [2]. It was reported by Monsarrat et al., [4] that after artificial aging, better surface integrity and less change were found in mechanical parameters for pure ormocers than for conventional ormocers and composites due to the elimination of the conventional methacrylate monomers. Also, polymer networks based on Bis-GMA are highly susceptible to chemical softening [29] which is why Grandio was affected more than Admira fusion. Moreover, the degree of conversion of dental composites is a fundamental criterion in determining the surface stability directly influencing the surface microhardness [30].

Regarding the surface roughness results, the change in surface roughness was the highest upon immersion in Pepsi Cola and it was the least in the control. Yet, all the surface roughness values were below the threshold for plaque retention (0.2 µm) [8].

Soft drinks may contain several different types of acids that can contribute to the low pH value such as the presence of phosphoric acid in cola. This was demonstrated to be highly erosive compared with other organic acids. Carbonated beverages contain also carbonic acid formed by carbon dioxide in solution. Even when the carbon dioxide has been blown off and drinks have become ‘flat’, the pH remains low. Such acidic pH was thought to be responsible for the increase in surface roughness of the tested resin composite [3]. This was in agreement with Hamouda [31] who reported that all restorative materials tested in his study became rougher after they had been subjected to the lower pH-cycling regimen.

Thermocycling is an in-vitro procedure in which the tested materials are exposed to significant temperature variations to imitate the oral cavity. The resin matrix and filler particles may experience different thermal volumetric changes because of different thermal expansion coefficients or thermal conductivity coefficients. It is also important to note that water sorption during heat cycling led to the hydrolytic breakdown of the bonding between the resin matrix and filler particles. Additionally, it was stated that hygroscopic expansion in the resin matrix and filler phase would occur concurrently with water sorption, hence accelerating the degradation between the filler and matrix. All these factors could be the route that led to the dislodgement of filler particles and might in part cause an increase in the surface roughness. In the current study, a typical thermocycling model was not performed. Instead, a practically simulating model was applied to imitate the clinical situation in which the used beverages were used at their consumption temperature and then returned to the artificial saliva at 37℃.

Brushing in the current study yielded an increase in surface roughness more than in the non-brushing groups. These results were in line with da Silva et al., [32], Roselino et al., [33], and Costa et al., [21]. Also in a study Paolone et al., [16] the use of toothpaste with high RDA produced detrimental effect on the nanohybrid composite used.

The change in surface roughness was less obvious in Grandio in most tested groups. The results were in agreement with Heintze et al., [34]. This could be explained by assuming that the large fillers were trimmed flat during brushing to compensate for the loss of the polymer matrix when the mean roughness is measured [34]. O’Neill et al., [35] in their study reported that Admira Fusion X-tra samples demonstrated the roughest surfaces after a 15,000 brushing cycle. This increase in surface roughness might be due to the presence of clumps of the pre-condensed inorganic filler that remained on the surface after the resin matrix had been brushed away [35]. It is worth noting that the wear behavior of composites is affected by other factors besides filler features, including monomer conversion of the resin matrix, the filler loading, and the quality of adhesion of the fillers to the matrix.

One of the limitations of this study is the difficulty in replicating the clinical situation with an in-vitro study. Also, the short period of evaluation is another limiting factor. In addition to that, only two dental composites were evaluated, and the effect of different finishing protocols was not evaluated.

## Conclusions

Given the limitations of this in-vitro study, it can be concluded that immersion of the two dental composites in staining solutions produced detrimental effect on their surface integrity increasing their surface roughness and decreasing their surface microhardness with red wine and soft drink producing a more pronounced effect. Moreover, using a high abrasive toothpaste produced a more profound decrease in the surface integrity of the two dental resin composites. The nanohybrid Ormocer offered no superior surface integrity compared to the nanohybrid composite.

## Data Availability

The datasets used and/or analyzed during the current study are available from the corresponding author upon reasonable request.
